# Incidence and outcome of severe ante-partum hemorrhage at the Teaching Hospital Yalgado Ouédraogo in Burkina Faso

**DOI:** 10.1186/s12873-017-0128-3

**Published:** 2017-05-31

**Authors:** Martin Lankoande, Papougnezambo Bonkoungou, Sosthène Ouandaogo, Marcelin Dayamba, Ali Ouedraogo, Francis Veyckmans, Nazinigouba Ouédraogo

**Affiliations:** 1 Resident Anesthesia and Resuscitation, Yalgado Ouedraogo Hospital, 15 BP 106 Ouagadougou 15, Burkina Faso; 2 Yalgado Ouédraogo Hospital, Ouagadougou, Burkina Faso; 3 Blaise Compaore Hospital, Ouagadougou, Burkina Faso; 4 Cliniques University Saint Luc, Woluwe-Saint-Lambert, Belgium

**Keywords:** Severe antepartum hemorrhage, Third trimester, Pregnancy

## Abstract

**Background:**

Hemorrhage is the main cause of maternal death during pregnancy. This study aims to evaluate incidence and outcomes of Severe Ante Partum Hemorrhage (SAPH) during the third trimester of pregnancy prior to delivery.

**Methods:**

Analytical cross-sectional study with prospective data collection during 12 months in Yalgado Ouedraogo Hospital, Ouagadougou, Burkina Faso. In this context SAPH is specifically referring to Ante Partum Hemorrhage (APH) and Intra Partum Hemorrhage (IPH) in the 3rd trimester. Postpartum Hemorrhage (PPH) was not included.

**Results:**

During our study 7,469 women were admitted in obstetrics and 122 cases of SAPH were recorded. SAPH represented 1.6% (*n* = 122) of hospitalizations causes and 14.5% (*n* = 1083) of hemorrhages during pregnancy. Mean age was 27.8 ± 6.9 years, mean parity 2.8 ± 1.3 and mean duration of pregnancy was 37 Weeks Amenorrhea (WA). Evacuation from other facilities was the main mode of admission (91.8%, *n* = 112) and blood transfusion was the essence of resuscitation. Complications were observed in 80.3% (*n* = 98). During the study, 118 maternal deaths were reported of which 15.6% (*n* = 19) related to SAPH. Among SAPH cases who died (*n* = 19) majority (*n* = 16) had severe anemia (*n* = 16; 82.6%, *p* = 0.004). Ten women (8.19%) were admitted in Intensive Care Unit (ICU). Fifteen premature births (12.3%) and 22 perinatal deaths (18.1%) were recorded. Evacuation (*p* = 0.04), critical clinical condition during admission (*p* = 0.004), and Uterine Rupture (UR) (*p* = 0.002) were associated with poor outcome. The Retroplacental Hemorrhage (RPH) (40.9%) was the most common cause of fetal death (*p* = 0.005) and was associated with High Blood Pressure (HBP) and pre-eclampsia.

**Conclusion:**

APH is a complication associated with significant maternal and fetal morbidity and mortality.

## Background

Worldwide approximately 830 women die every day from preventable causes related to pregnancy and childbirth; 99% of those 830 daily deaths are women from developing countries. [1] Direct obstetrical complications are the main causes of maternal deaths, with bleeding [2] identified as the first cause. In Burkina Faso maternal mortality remains high, with 2,700 deaths per 100,000 deliveries [1]. Hemorrhage is the leading cause of maternal mortality globally, accounting for approximately 27% of deaths worldwide; this includes postpartum, intrapartum and antepartum hemorrhage [3]. In developed countries, hemorrhage prior to delivery accounts for only 16.3% of maternal deaths, while Sub-Saharan Africa remains high at 24.5%. Antepartum Hemorrhage (APH) is defined as bleeding from the vagina after 24 weeks [4]. In France [5] Serious Obstetrical Hemorrhage (SOH) represented 19% of admissions to the Intensive Care Unit (ICU). Worldwide postpartum hemorrhage (PPH) has been well studied, but APH and intrapartum hemorrhage (IPH) are less well-documented. APH is an obstetric emergency significantly contributing to perinatal and maternal morbidity and mortality. PPH yields mainly maternal complications and may be in itself a complication of APH. However, during APH, complications can be fetal as well as maternal. The maternal complications are malpresentation, premature labor, PPH, sepsis, shock and retained placenta [6]. Various fetal complications are prematurity, low birth weight, intrauterine death, congenital malformation and birth asphyxia [7].

The obstetric department of Ouagadougou is the main and sole national reference center. Patients are generally referred from health facilities at least 150 km from Ouagadougou. The obstetric department has 81 beds plus 9 intensive care beds. It is poorly equipped in material, and drug supply is scarce. Specialized personnel consist of 13 obstetricians, 17 nurses specialized in anesthesia, 38 midwifes, and one anesthesiologist. Our study aims to evaluate the incidence and outcome of severe APH in the third trimester of pregnancy prior to delivery in Yalgado Ouedraogo Hospital in Burkina Faso.

## Methods

This was an analytical cross-sectional study with prospective collection of data during 12 consecutive months (1^st^ of February 2012 to 31^st^ of January 2013). Informed written consent was obtained from each participant or her guardian, and confidentiality was assured. Patients who refused to participate were thanked without any further pressure to change or explain their refusal. The study included women admitted in the obstetric service during their third trimester of pregnancy with hemorrhage. To qualify as “Severe Ante-Partum Hemorrhage” in this study, the hemorrhage had to satisfy at least one of the following criteria: a blood loss above 2000 ml, a loss of 150 ml/min, a loss of 50% of blood volume within 3 h, a decrease of maternal hemoglobin concentration of 4 g/dl, transfusion needs greater than 4 units, [8] maternal hemoglobin concentration less than 6 g/dl at any point of time, bleeding with vital signs instability (maternal pulse less than 50 or higher than 100 bpm, systolic blood pressure less than 100 mmHg and diastolic pressure ≤ 50 mmHg) or hemorrhagic shock. In our study, serious obstetrical hemorrhage is specifically referring to APH and IPH in the 3^rd^ trimester. Patients who met at least one of these criteria were included. Our center already published its results about PPH, which results are therefore not included in the present study. Data collection was based on the clinical review, interview, admission register and ultrasound archives. Causes of hemorrhage were diagnosed by ultrasound, clinical assessment or intra-operative findings. Socio-demographic data, causes for hemorrhage, case management, maternal and fetal outcomes were reviewed and analyzed. Delay to transfusion was defined as the time elapsed from the decision to transfuse to actual blood transfusion. Definitions: A transfer is the referral of a patient from another service in the same hospital. A reference is the non-emergency transfer of a patient from a health facility to another high performance facility. An evacuation is an emergency referral of a patient from a health facility to another high performance facility. Data were analyzed with EPI Info 3.5 and results expressed as a mean for quantitative values, median, extremes and a percentage for qualitative values. Association between variables was investigated by the Chi2 test, and a p value less than 0.05 was considered as statistically significant. The present study was approved by National Center of Scientific Reseach and Technologic (NCSRT) committee of Burkina Faso. Strict confidentiality was ensured for all personal data.

## Results

During our study, 7,469 women were admitted in obstetrics. Among them, 3,645 women experienced a hemorrhage, and from that group again 122 (1.6%) women experienced SAPH. Seventy-nine patients live inside Ouagadougou. Demographic, obstetrical and clinical data are illustrated in Tables 1 and 2 below. Table 2 focuses on clinical data and the causes of hemorrhage.

**Table 1:** Demographic and obstetrical data

Data	Mean ± α [95%]	Number n	Frequency %
Age (years)	27.8 ± 6.9 [16–45]		
Parity	2.8 ± 1.3 [0–8]		
Job			
Housewife		82	67.2
Official		9	7.4
Informal sector		12	9.8
Scholar/Student		19	15.6
Mode of admission			
Referral from others facilities		112	91.8
Direct admission		9	7.4
Already hospitalized		1	0.8
Age of pregnancy	37 WA [28–41]		
≥3 Prenatal Consults		92	75
Pregnancy-related morbidity		98	80.3
Hemorrhage		22	22.5
Anemia		89	90.8
Systemic Hypertension		16	16.3
Pre-eclampsia		27	22.13
History of Cesarian section		7	5.7

*WA* Weeks of amenorrhea

**Table 2:** Clinical and biological data (*n* = 122)

Data	Mean ± α [95%]	Number n	Percentage %
Admission diagnosis			
Hemorrhage		78	63.9
Pelvic pain		12	9.8
Anemia		28	23,0
Coma		4	3,3
Condition during admission			
Critical condition		48	39.4
Stable condition		74	60.6
Hb prior to transfusion order	6.8g/dl ± 1,2		
≤ 6g/dl		101	82.8
6-9g/dl		16	13.1
9-11g/dl		5	4.1
Classification			
Antepartum		78	63.9
Intra partum		44	36.1
Causes of hemorrhage			
Placenta Prævia		52	42.6
Retro Placental Hematoma		40	32.8
Uterine Rupture		30	24.6

*Hb* Hemoglobin concentration

In our study, 112 (91.8%) patients were evacuated. From that total, 63 patients (56.3%) were from inside the capital city of Ouagadougou and 49 patients (43,7%) were from outside Ouagadougou. During evacuation, 65% of all women evacuated were accompanied by a nurse, none by a doctor. Women coming from outside Ouagadougou (35.2%) were assisted by nurse only (18%) and others came by themselves (4.5%). Retroplacental hematomas occurred much more frequently in patients with high blood pressure (HBP) history (75%, *n* =12/16; *p* = 0,004) or pre-eclampsia (96.3%; *n* = 26/27 *p* = 0,002). Hemorrhage and pelvic pain (73.7%) were the main causes of admission.

**Fig. 1:**
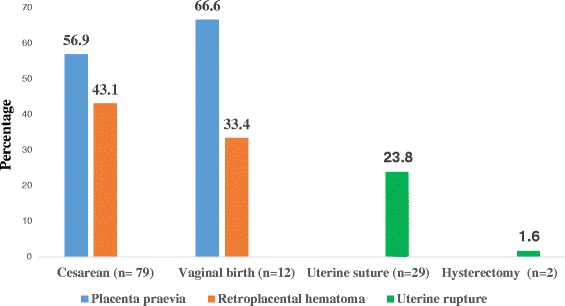
Obstetrical management data (*n* = 122). The Figure 1 describe the obstetrical management of serious ante partum hemorrhage according type of pathology. There are three types of diseases as placenta previa, retroplacental hematoma uterine rupture. These diseases are managed by cesearan section mainly (*n* = 110) againts vaginaly birth in 12 cases. Among cesearan cases, uterine suture was performed in 29 cases against hysterectomy in two cases

The following treatments were applied: left lateral position, oxygen supplements (*n* = 78; 63.9%), perfusion of crystalloids and transfusion. Fourteen patients (*n* = 14; 11.5%) received no transfusion before the 24^th^ hour of their hospitalization. Blood transfusion data are described in Table 3 below. Most patients received antibiotics (*n* = 114; 93.4%) and oxytocin (*n* = 82; 67.2%). Cesarean delivery was performed in 100 patients and 12 by vaginal delivery. Among cesarean cases (*n* = 79), 29 wises had uterine suture and 2 had hysterectomy because of uterine rupture. Specific maternal mortality was higher in vaginal delivery (75%; *n* = 10; *p* = 0,004) than Cesarean delivery (*n* = 9; 9.1%; *p* = 0.1). Obstetrical management is described in Fig. 1. No patient benefited from interventional radiology because of its unavailability in Burkina Faso. Ten patients (8.19%) were admitted in the Intensive Care Unit (ICU) where six of them died. Resuscitation consisted of oxygen therapy, intravenous fluids administration, transfusion, vasoactive drugs (5 cases), antibiotics, intubation and mechanical ventilation (3 cases). Complications occurred for 80.3% (*n* = 98) of patients including worsening anemia (90 cases, 73.7%), severe sepsis (*n* = 26; 21.3%) and hemostatic disorders (*n* = 4, 3.2%). Table 4 below describe maternal and fetal outcomes.

**Table 3:** Blood transfusion data (*n* = 122)

Blood transfusion	Mean value	Number n	Percentage %
Transfusion		103	84.4
Erythrocytes		101	82.9
Fresh frozen plasma		2	1.6
Unavailability of blood		19	15.6
Transfused Volume	450 ml ± 96		
Delay of transfusion	3.8 h ± 1.7		
Hemoglobin level after transfusion	6.8 g/dl

**Table 4:** Maternal and fetal outcomes (*n* = 122)

Variables	Mean	Number n	Mortality n	Mortality %	*p*
Admission					
External transfer		112	19	16.9	0.004
Self-consultation		9	0		
Internal Referral		1	0		
Clinical status					
Critical		48	12	25	0.002
Stable		74	7	5.7	
Age (years)	27.2				0.3
[16–20]		25	6	24	
[21–25]		31	4	12.9	
[26–30]		30	2	6.7	
[31–35]		21	5	23.8	
[36–40]		14	2	14.3	
[41–45]		1	0	0	
All		122	19	15,6	
Etiology					
Retroplacental hematoma		40	7	17.5	0.04
Placenta Previa		51	3	5.8	
Uterine Rupture		31	9	29	0.002
Maternal outcome					
ICU and transfused		10	10	100	0.3
Transfused in obstetric		93	4	4.3	0.56
Not transfused		9	5	55.55	0,001
Evacuated		112	16	14,3	0,003
Not evacuated		10	3	30	0,08
Cesarean delivery		110	10	9,1	0,1
Vaginal delivery		12	9	75	0,004
Fetal outcome					
Evacuated		112	21	18,7	0,0001
Not evacuated		10	1	10	0,07
Cesarean delivery					
Placenta Previa		45	2	4.44	0.01
Retroplacental hematoma		34	5	14.28	0.005
Uterine Rupture		30	6	20	0,03
Vaginal birth					
Placenta Previa		8	5	62.5	0.01
Retroplacental hematoma		4	4	100	0.003
Uterine Rupture		–	–	–	–

Our study showed SAPH mortality rate was 16.1%. The average age of deceased patients was 27.2 years and the majority of them were evacuated. Reference (*p* = 0,001) and not transfusion (*p* = 0,002) were risks factors associated with maternal mortality. Patients died because of multiple organ failures in eight cases, and coagulopathy (*n* = 6) or lack of blood products. Preterm delivery occurred in 12.3% (*n* =15). Twenty-two perinatal deaths (18.1%; *n* = 22) were recorded, including 12 apparent stillborn and 10 macerated stillborn. Retro placental hematoma was associated with a 25% fetal mortality rate (*n* = 10/40 cases), significantly higher than placenta praevia (*n* = 7 cases on 52, 13.5%) and uterine rupture (*n* = 6/30, 20%) (*p* = 0.005) as indicated in Table 4 below.

## Discussion

SAPH are not very frequent in our study (1.6%). Such low frequency also was reported by Takpara [9] et al. (5.5%) in Benin. In comparison to nearly 5% of pregnancies [10] in France. In our study, 78 patients had ante partum hemorrhage (63.9%) against 44 cases (33.1%) for intra partum hemorrhage. According to a World Health Organization (WHO) study in Sub-Saharan Africa, APH accounts for 8.4% of all maternal hemorrhage versus 0.9% for IPH^3^. SAPH is more frequent in developing countries than in developed countries [3, 11] In fact, data must be analyzed cautiously; our study is limited to severe hemorrhage while most publications concern all types of hemorrhage.

The average age of our patients was 27.8 years. In Burkina Faso, half of women marry before 17.5 years old, and the majority of women give birth to their first child before 20 years old [12]. This young age is reported by other authors, [13, 14] Women living outside Ouagadougou represented 35.2% of total patient our study. These patients were referred which contributed to a delay in decision making and increased maternal morbidity and mortality. During admission, severe clinical anemia (≤6 g/dl) was observed in 101 patients (82.8%).

The majority of women were referred form other facilities (91.8%). Pregnancies had been relatively well attended with 3 antenatal consultations for 65% of them. Burkina Faso has a pyramidal organization of health care facilities. The obstetric department of Ouagadougou is the main and only national reference center. Patients are generally referred from health care facilities located more than 150 km from Ouagadougou. In our study, 112 (91.8%) patients were referred. During reference, 65% of all women referred were accompanied by a nurse, none by a doctor. Women coming outside Ouagadougou (43,7%) were assisted by nurse only (18%) and others came themselves (4.5%). Ouattara and al [15] reported that the reference process accounted for 43% of admissions in the same service where we conducted our study. The insufficiency of heath service and poorly maintained or low availability of equipment are some of explanations of this situation. Women were relatively followed up during pregnancy in our study like some other study [12].

There was a delay in the management of pregnant patients’ bleeding and patients were often previously anemic. Rhesus blood grouping and hemoglobin measurement were performed in emergency as described in the literature [9, 16] Given the excellent results of ultrasound examination, any suspicion of obstetric pathology justifies its use, but because of unavailability, it was performed only for 6 patients (5%), identifying 4 cases of Placental Previa (PP) and 2 cases of Retroplacental hematoma (RPH) in our study. The main causes of SAPH are PP, followed by RPH and Uterine Rupture (UR). These three pathologies are common in Africa [9, 14] The PP incidence accounted for 41.80% of severe APH (*n* = 51) in our series. In the literature, abnormal placental insertions were responsible of 5 to 10% of SAPH [10, 17] In our study, RPH caused 32.8% (*n* = 40) of SAPH. Takpara et al. [9] in Benin reported that RPH was responsible of 5.8% of serious obstetrical hemorrhage. RPH occurred in 0.5% of all pregnancies, and complicate 0.2 to 0.4% of these pregnancies with a perinatal mortality about 20.2% related to hemorrhage [17, 18] Obstetric hemorrhage was due to UR in 42% (*n* = 31) in our study from which 2 cases (6.5%) occurred on a scarred uterus and other ¾ on healthy uterus. In our study, the high proportion of UR on healthy uterus may be related to multiparty, lack of support for pregnancy, fetal and pelvic disproportion, unfavorable socio-economic status, prolonged labor, and delayed consultation.

SAPH was responsible of 8.2% of intensive care unit admissions in our study. All cases of SAPH must be admitted in intensive care, but the lack of health insurance is an obstacle to their admission unlike in others countries [5, 18, 19] Despite the availability of Emergency Obstetric and Neonatal Care (EmONC), there was no protocol for hemorrhage management in this service. Measures such as the left lateral position, oxygen supplement were effective for 77 patients (63.4%). Volume expansion with saline (*n* = 122) and gelofusin (*n* = 88; 72%) were implemented. Thirteen percent (*n* = 16) of women did not receive oxygen due to lack of supplies (oxygen mask, nasal prongs). Like in others’ Africans studies, maternal resuscitation was hampered by the lack of blood products [20]. In our studied population, uncovered transfusion needs were reported for 18 patients (14.8%), but no patient needed massive transfusion. The average waiting time for transfusion was long (3.8 h) and Bonkoungou [21] in the same service reported 5.2 h delay to transfusion. In our series, one of the ten women died because of the lack of blood products. The transfusion products were mainly erythrocytes. Two patients received fresh frozen plasma for clotting disorders. There are no publications on stock-outs of supplies and blood in this Hospital and data are unavailable. A further research information is needed to establish the no covered rate of blood transfusion. Oxytocin and antibiotics were used in our study like in others studies [9].

A Cesarean section was performed for 79 patients (64.8%). This rates are similar to Beninese data [9]. Eight woman who gave birth vaginally could have been cared in a peripheral clinic and this raises questions about the qualification of agents that referred women. A hysterectomy was performed in 2 cases (1.6%) after failure of conservative treatment. In the study of Nayama et al. [22] in Niger 18,934 deliveries had been registered from which 154 patients had benefited from obstetric hysterectomy (0.8%). Despite this radical rescue surgery, a patient died in post-operative period. The decision to perform a hysterectomy remains difficult, especially in the African socio-cultural context [13] and among young women. The delay in management explains the high mortality reported both in our study and in the Takpara [9] study.

The major morbidity was anemia (73.4%) in our study as described in the literature [14]. Anemia is often part of the continuum of pregnancy. The second cause of morbidity was infection. Maternal death rate in our study was 15.6%. Hemorrhage is responsible for 27,1% of maternal deaths according to Say and al [3]. The mortality rate in our series is comparable to what Cissé [23] reported (16.4%). UR was the deadliest (29%) (*p* = 0.06) in our study. According to literature RPH caused 7 to 20% of maternal deaths related to hemorrhage during the third trimester of pregnancy [17, 18]. The need of patients to be referred (*p* = 0.04), critical condition during admission (*p* = 0.004), and uterine rupture (*p* = 0.002) were poor outcome factors in our study. In Africa, the insufficiency of equipment, drugs for resuscitation and delay to health care utilization increase the mortality rate. So delay can be attributed to poor access to supplies and to blood products. Despite resuscitation and Cesarean section practice, fetal outcome remained severe. Fifteen premature births (12.3%) and 22 perinatal deaths (18.1%) were recorded in our study. This rate of stillbirths was comparable to Takpara [9] data (14.7%). RPH remained the most feticide (27.3%) in our study. According to Mercier and al [24] RPH was responsible of 20.2% perinatal mortality. During pregnancy, high blood pressure, pre-eclampsia must be researched and managed in order to reduce risk of placenta abruption.

## Conclusion

APH is serious obstetric complication. Young women were concerned. SOH is associated with significant maternal and fetal morbidity and mortality. It is a frequent reason for consultation, and its outcome can be fatal without early and appropriate management. The lack of blood products, insufficient equipment, poor medical condition upon transfer from health facilities, and delayed resuscitation each impacted the effectiveness of the APH management. Since April 2016 with the establish 43,7%) ent of general health insurance for obstetric and pediatric care in our country, we hope that pregnancy will have more attention and good care. However, some effort must be done to improve availability of skilled resources.

## Abbreviations

EmONC: Emergency obstetric and neonatal care
HBP: High blood pressure
ICU: Intensive care unit
IPH: Intra partum hemorrhage
PC: Prenatal consultation
PP: Placenta previa
PPH: Postpartum hemorrhage
RPH: Retroplacental hematoma
SAPH: Severe antepartum hemorrhage
UR: Uterine rupture
WA: Weeks of amenorrhea
